# Continuous positive airway pressure in acute ischemic stroke patients with obstructive sleep apnea: analysis of the National Inpatient Sample database

**DOI:** 10.3389/fneur.2025.1624631

**Published:** 2025-11-04

**Authors:** Yaqing Zeng, Chaoyang Zheng, Mei Qi, Hui Wang, Jianli He, Xiaoqiang Li, Hao Xie, Qin Wang

**Affiliations:** ^1^Department of Neurology, Xiaolan People's Hospital of Zhongshan (the Fifth People's Hospital of Zhongshan), Zhongshan, Guangdong, China; ^2^Department of Pediatric Orthopedic, Center for Orthopedic Surgery, the Third Affiliated Hospital of Southern Medical University, Guangzhou Guangdong, China; ^3^Division of Orthopaedic Surgery, Department of Orthopaedics, Nanfang Hospital, Southern Medical University, Guangzhou, Guangdong, China

**Keywords:** obstructive sleep apnea, acute ischemic stroke, continuous positive airway pressure, outcomes, Nationwide Inpatient Sample, routine discharge

## Abstract

**Background:**

Obstructive sleep apnea (OSA) is an established independent risk factor for stroke. However, the efficacy of continuous positive airway pressure (CPAP) in patients with acute ischemic stroke and comorbid OSA (AIS-OSA) remains uncertain. This study aimed to assess the impact of CPAP on hospitalization outcomes for AIS-OSA patients using data from the National Inpatient Sample (NIS).

**Methods:**

A retrospective data analysis was conducted using the NIS to identify patients hospitalized with a diagnosis of AIS-OSA between 2010 and 2019 with complete data. Patients were categorized into two groups based on CPAP treatment during hospitalization. Logistic regression analyses were performed to identify factors associated with CPAP treatment.

**Results:**

Among 103,004 patients with AIS-OSA, those who received CPAP had statistically significant longer lengths of stay (LOS), higher medical expenses, and increased in-hospital mortality rates. Conversely, this group also exhibited higher proportions of routine discharges, suggesting potentially improved long-term outcomes. Independent predictors for CPAP treatment included advanced age, Black race, congestive heart failure, and obesity. Besides, factors such as pneumonia, acute myocardial infarction, pulmonary embolism, intracranial hemorrhage, thrombolysis, and mechanical thrombectomy may be associated with CPAP treatment, but this association does not imply causation.

**Conclusion:**

This study identified independent predictors and associated factors for CPAP treatment in hospitalized AIS-OSA patients. Our observation may suggest that surviving patients who received CPAP treatment had more favorable prognoses, however, randomized trials are needed to determine causality.

## Introduction

Sleep apnea, a prevalent chronic disease, is recognized as an independent risk factor for stroke, cardiovascular disease, and all-cause mortality (1, 2). It has been shown to increase the incidence rate of acute ischemic stroke (AIS) and is associated with worse functional outcomes and higher stroke recurrence rates (3, 4). More than half of stroke survivors exhibit sleep apnea during the acute post-stroke phase, with obstructive sleep apnea (OSA) being the most prevalent subtype (5, 6). The pathophysiology linking OSA to stroke remains unclear (3). Given that stroke and OSA are intertwined conditions with shared risk factors and comorbidities (7), OSA poses additional challenges in the acute-phase management of stroke patients. Continuous positive airway pressure (CPAP) is the gold standard for OSA treatment, targeting both anatomical and non-anatomical mechanisms (8). Some studies suggest that OSA therapy is feasible and may improve long-term outcomes in stroke patients (9–12), while others indicate that CPAP treatment could also aid in stroke prevention (13, 14). However, due to limited evidence in the acute phase, there are no clear clinical guidelines for this patient population.

Understanding the epidemiology and outcomes of patients with acute ischemic stroke and comorbid obstructive sleep apnea (AIS-OSA) is crucial for identifying subgroups that may benefit from CPAP and for recognizing unmet needs within this patient population.

This study analyzed the prevalence, comorbidities, complications, and outcomes of hospitalized patients with AIS-OSA using data from the National Inpatient Sample (NIS). Furthermore, the outcomes of AIS-OSA patients who received CPAP during hospitalization were examined.

## Materials and methods

### Data source

This retrospective study utilized the NIS, a Healthcare Cost and Utilization Project (HCUP) database sponsored by the Agency for Healthcare Research and Quality (AHRQ). The NIS is an all-payer database that approximates a 20% stratified sample of discharges from community hospitals in the United States (15, 16).

The NIS database was retrieved for data on hospitalizations from 2010 to 2019. The NIS database comprises an identified collection of procedural and diagnostic codes from participating hospitals. As the NIS dataset does not directly involve human subjects (consistent with federal regulations and guidance), it is exempt from institutional review board approval.

### Inclusion and exclusion criteria

**Figure 1:**
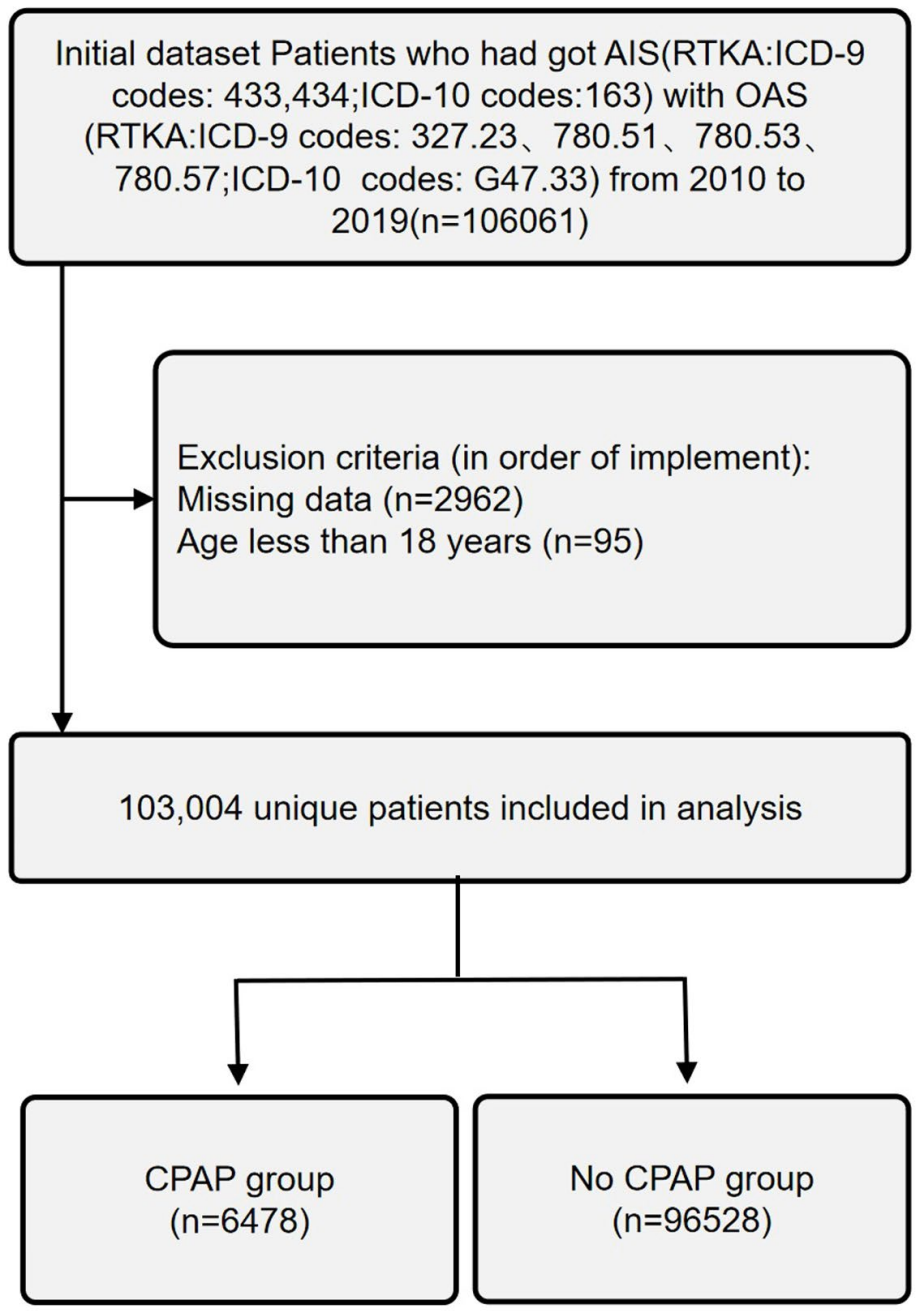
Exclusion process for AIS-OSA patients undergoing CPAP.

All patients aged 18 years or older, admitted to the hospital with a primary diagnosis of AIS and OSA, were included in this study. AIS was identified using the International Classification of Diseases, Ninth Revision, Clinical Modification (ICD-9-CM) codes 433 and 434, and the International Classification of Diseases, Tenth Revision, Clinical Modification (ICD-10-CM) code I63. OSA was identified using ICD-9-CM codes 327.23, 780.51, 780.53, and 780.57, and ICD-10-CM code G47.33. Cases with a primary diagnosis code other than AIS and OSA were excluded. Patients with other forms of stroke were also excluded. Baseline demographic data, including age, race, sex, number of comorbidities, type of admission (non-elective, elective), hospital bed size, hospital teaching status, hospital location, type of insurance, discharge disposition, length of stay (LOS), in-hospital mortality, and total hospital charges (TOTCHG), were extracted. Clinical variables, representing the known history of Acquired Immune Deficiency Syndrome (AIDS), alcohol abuse, deficiency anemia, rheumatoid diseases, chronic blood loss anemia, congestive heart failure, chronic pulmonary disease, coagulopathy, depression, diabetes (uncomplicated), diabetes (with chronic complications), drug abuse, hypertension, hypothyroidism, liver disease, lymphoma, fluid and electrolyte disorders, metastatic cancer, neurological disorders, obesity, paralysis, peripheral vascular disorders, psychoses, pulmonary circulation disorders, renal failure, solid tumor without metastasis, peptic ulcer disease, valvular disease, and weight loss, were obtained using ICD-9-CM and ICD-10-CM diagnosis codes (Table 1). Patients were categorized into two groups: CPAP and no-CPAP, based on the presence of ICD-9-CM procedure code 93.90 and ICD-10-CM procedure codes 5A09357, 5A09457, and 5A09557. Patients younger than 18 years or those with missing data were excluded from this research (Figure 1).

**Table 1:** Variables used in binary logistic regression analysis.

Variables categories	Specific variables
Patient demographics	Age (≤64 years and ≥65 years), sex (male and female), race (White, Black, Hispanic, Asian or Pacific Islander, Native American and Other), number of Comorbidity
Hospital characteristics	Type of admission (non-elective, elective), bed size of hospital (small, medium, large), teaching status of hospital (nonteaching, teaching), location of hospital (rural, urban), type of insurance (Medicare, Medicaid, private insurance, self-pay, no charge, other), location of the hospital (northeast, Midwest or north central, south, west), disposition (routine, transfer to short-term, hospital, transfer other, HHC, AMA, died in hospital, discharged alive), TOTCHG, LOS
Comorbidities	AIDS, alcohol abuse, deficiency anemia, rheumatoid diseases, chronic blood loss anemia, congestive heart failure, chronic pulmonary disease, coagulopathy, depression, diabetes (uncomplicated), diabetes (with chronic complications), drug abuse, hypertension, hypothyroidism, liver disease, lymphoma, fluid and electrolyte disorders, metastatic cancer, neurological disorders, obesity, paralysis, peripheral vascular disorders, psychoses, pulmonary circulation disorders, renal failure, solid tumor without metastasis, peptic ulcer disease, valvular disease and weight loss

AIDS, acquired immunodeficiency syndrome.

### Outcomes

Outcomes assessed in this study included discharge disposition, in-hospital mortality, TOTCHG, LOS, and in-hospital complications. In-hospital complications were categorized as neurological complications (seizure, intracranial hemorrhage, receipt of thrombolysis, receipt of mechanical thrombectomy) and medical complications (sepsis, acute myocardial infarction, deep vein thrombosis, pulmonary embolism, cardiac arrest, acute kidney injury, pneumonia, urinary tract infection).

### Statistical analysis

Patients with AIS-OSA were identified using ICD-9 and ICD-10-CM codes. This cohort was subsequently stratified into two groups based on the administration of CPAP therapy during hospitalization. Continuous variables are presented as median and interquartile range (IQR). Categorical variables are presented as frequencies and percentages. Comparisons of demographics, clinical characteristics, and outcomes between the groups were conducted using the Mann–Whitney *U* test for continuous variables and the chi-square test or Fisher’s exact test, as appropriate, for categorical variables. Regression analyses were performed to identify statistically significant factors associated with CPAP therapy and to evaluate the association between CPAP therapy and the specified outcomes, after adjusting for identified confounding variables. All statistical comparisons were two-tailed, and statistical significance was defined as a *p*-value less than 0.05 (*p* < 0.05). Statistical analyses were performed using SPSS software, version 25 (IBM Corp., Armonk, NY).

## Results

### Incidence of CPAP in AIS-OSA patients

**Figure 2:**
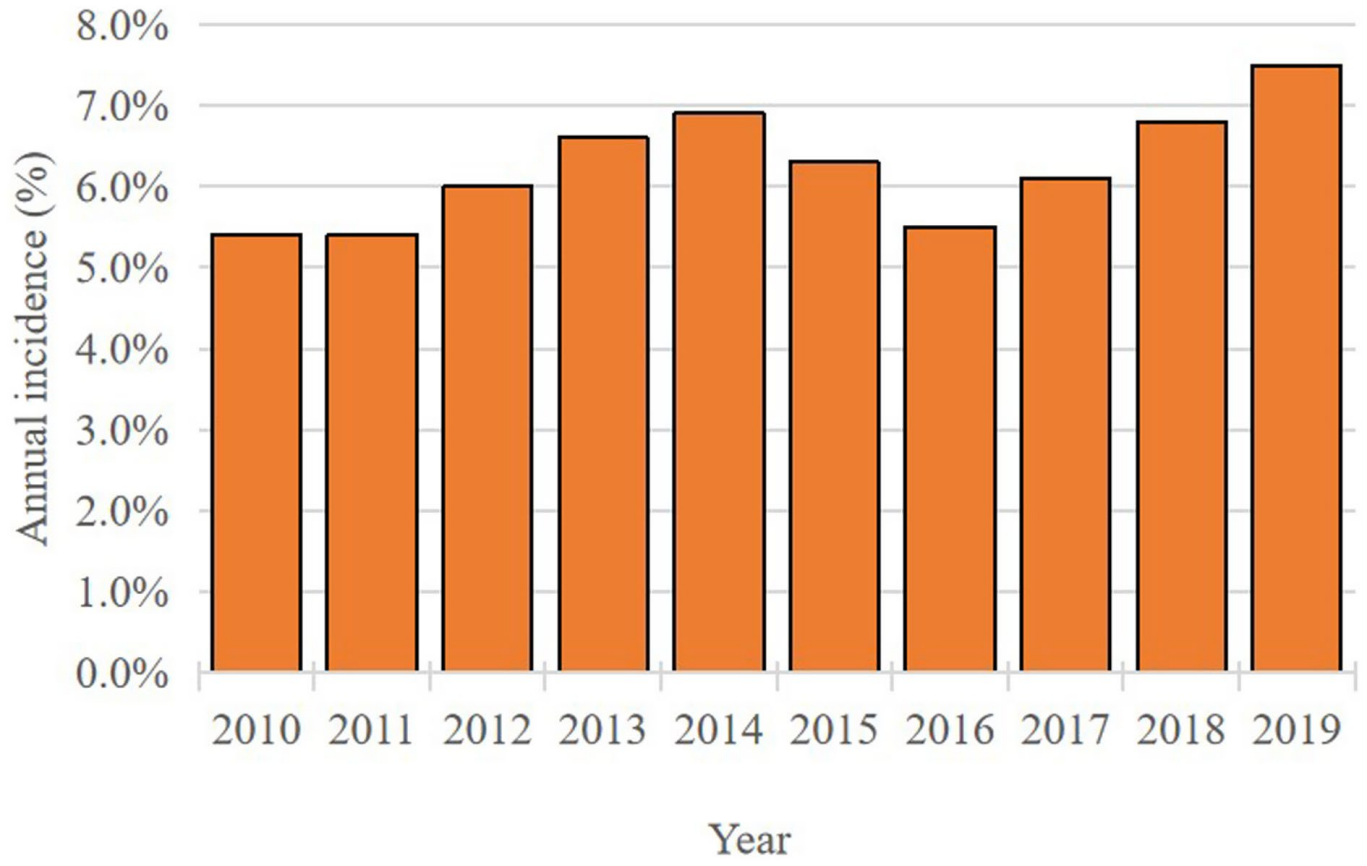
Annual incidence of CPAP in AIS-OSA patients.

**Figure 3:**
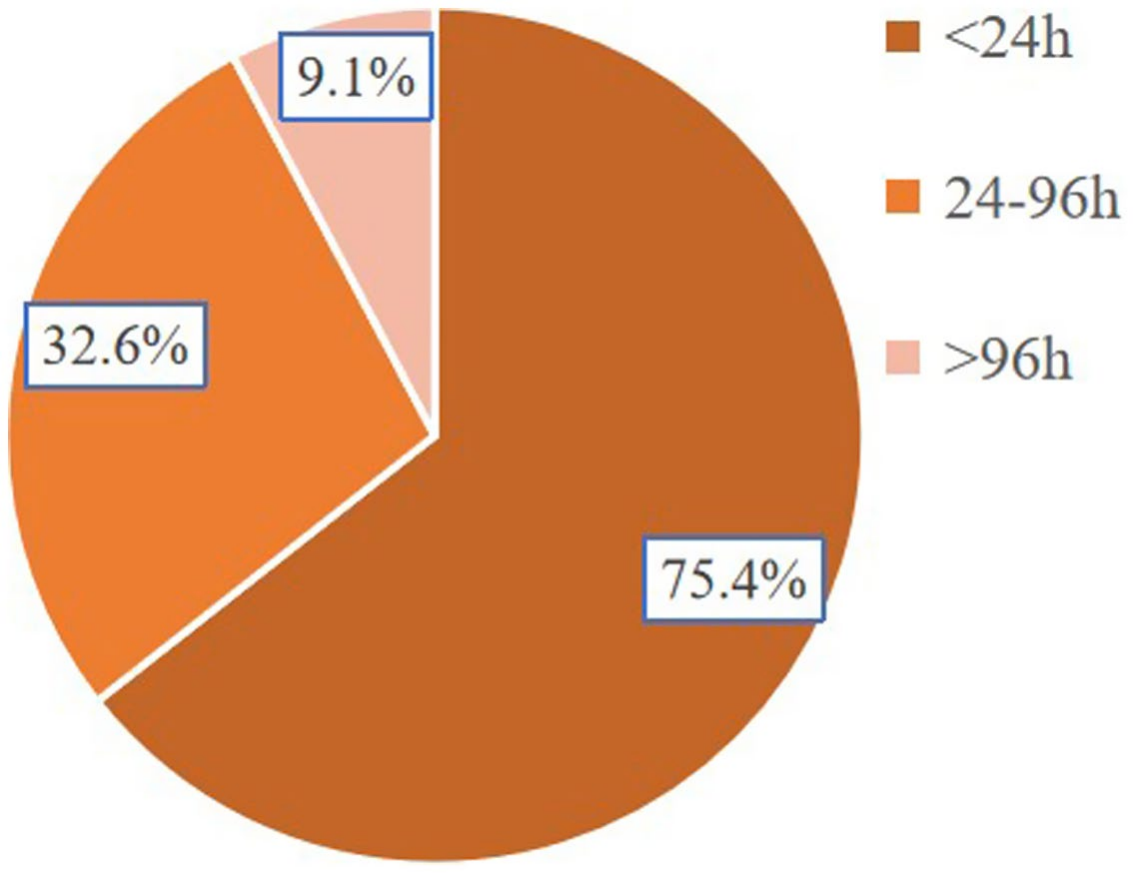
The proportion of patients with different CPAP treatment durations.

Between 2010 and 2019, a total of 106,061 patients with AIS-OSA were identified in the NIS database. Following the exclusion of patients who did not meet the prespecified inclusion criteria, a final cohort of 103,004 AIS-OSA patients was included in the analysis. Among these, 6,478 patients received CPAP treatment during hospitalization, yielding a CPAP utilization rate of 6.3% (Table 2). The data showed that the utilization rate of CPAP remained relatively consistent throughout the study period from 2010 to 2019 (Figure 2). Among AIS-OSA patients who received CPAP treatment, 75.4% were treated for less than 24 h, 15.6% for 24–96 h, and 9.1% for more than 96 h (Figure 3).

**Table 2:** AIS-OSA patient and hospital characteristics (2010–2019).

Characteristics	CPAP	No CPAP	*p*
Total (*n* = count)	6,476	96,528	
Total incidence (%)	6.3%		
Age (median, years)	68 (60,76)	69 (60, 77)	0.016
Age group (%)			
18–44	4.4	3.5	0.002
45–64	33.2	33.3	
65–74	32.6	32.5	
≥75	29.8	30.7	
Gender (%)			
Male	63.5	62.7	0.163
Female	36.5	37.3	
Race (%)			
White	71.2	73.9	<0.001
Black	15.2	12.1	
Hispanic	5.6	4.5	
Asian or Pacific Islander	1.7	1.1	
Native American	0.4	0.4	
Other	5.9	8.0	
Number of comorbidity (%)			
0	0.3	1.0	<0.001
1	3.1	6.0	
2	8.4	13.5	
≥3	88.1	79.5	
LOS (median, d)	6 (3–11)	4 (2–7)	<0.001
TOTCHG (median, $)	62003.0 (31820.3–124985.8)	37725.5 (21830.0–71796.0)	<0.001
Type of insure (%)			
Medicare	65.7	66.8	<0.001
Medicaid	8.0	6.3	
Private insurance	21.4	22.2	
Self-pay	2.0	2.3	
No charge	0.1	0.2	
Other	2.8	2.2	
Bed size of hospital (%)			
Small	12.4	13.6	0.001
Medium	25.1	26.2	
Large	62.5	60.3	
Elective admission (%)	12.1	16.6	<0.001
Type of hospital (teaching %)	67.5	62.6	<0.001
Location of hospital (urban, %)	93.8	92.0	<0.001
Region of hospital (%)			
Northeast	19.8	13.2	<0.001
Midwest or North Central	25.3	30.1	
South	38.4	40.1	
West	16.6	16.6	
Disposition			
Routine	43,773 (45.3%)	1,985 (30.7%)	0.001
Transfer to short-term hospital	2,771 (2.9%)	227 (3.5%)	
Transfer other	31,863 (33.0%)	2,760 (42.6%)	
Home Health Care (HHC)	14,284 (14.8%)	1,028 (15.9%)	
Against medical advice (AMA)	474 (0.5%)	25 (0.4%)	
Died in hospital	446 (6.9%)	3,325 (3.4%)	
Discharged alive	38 (0.0%)	5 (0.1%)	

LOS, length of stay; TOTCHE, total charge.

### Demographics and hospital characteristics

Patients who received CPAP were statistically significantly younger compared to those who did not (median age 68 years vs. 69 years, *p* = 0.016). The age distribution also differed statistically significantly between the two groups, with the CPAP group exhibiting a higher proportion of patients aged 18–44 years (4.4% vs. 3.5%) and 65–74 years (32.6% vs. 32.5%, *p* = 0.002) (Table 2). Furthermore, a statistically significant difference was observed in the race distribution: Black (15.2% vs. 12.1%), Hispanic (5.6% vs. 4.5%), and Asian or Pacific Islander (1.7% vs. 1.1%) patients comprised slightly larger proportions in the CPAP group (*p* < 0.001) (Table 2). AIS-OSA patients treated with CPAP were more likely to have more than three comorbidities (88.1% vs. 79.5%, *p* < 0.001). Moreover, patients receiving CPAP had a statistically significantly higher likelihood of using Medicaid as their primary insurance (8.0% vs. 6.3%, *p* < 0.001) (Table 2).

The geographic distribution of patients exhibited marked variability. A greater proportion of AIS-OSA patients receiving CPAP were treated at large hospitals (62.5% vs. 60.3%, *p* = 0.001), hospitals located in the Northeast region (19.8% vs. 13.2%, *p* < 0.001), and urban hospitals (93.8% vs. 92.0%, *p* < 0.001). Most patients in both cohorts were treated at teaching hospitals (67.5% vs. 62.6%, *p* < 0.001). Notably, AIS-OSA patients receiving CPAP treatment were less likely to have elective admissions (12.1% vs. 16.6%, *p* < 0.001). The baseline characteristics of the study cohorts are summarized in Table 2.

### In-hospital quality measures and disposition

Patients with AIS-OSA who received CPAP exhibited a statistically significant increase in the median LOS (6 days [IQR, 3–11 days] vs. 4 days [IQR, 2–7 days], odds ratio [OR]: 2.68 (95% confidence interval [CI], 2.6–2.8), *p* < 0.001) compared to patients who did not receive CPAP. Consequently, CPAP treatment was associated with elevated medical expenditures. Specifically, the median hospitalization costs for patients receiving CPAP were substantially higher, with total hospital charges amounting to $62,003 (IQR, $31,820–$124,986) compared to $37,726 (IQR, $21,830–$71,796) for those without CPAP (*p* < 0.001) (Table 2). Among patients who survived to discharge, those with AIS-OSA who received CPAP had a statistically significantly greater proportion discharged to routine care (45.3% vs. 30.7%, *p* < 0.001) (Table 2). Furthermore, among all AIS-OSA patients, CPAP treatment during hospitalization was found to statistically significantly increase the odds of in-hospital mortality (6.9% vs. 3.4%, *p* < 0.001) (Table 2).

### Risk factors associated with CPAP in AIS-OSA patients

Logistic regression analysis was employed to identify risk factors associated with CPAP treatment, revealing the following statistically significant predictors: advanced age (≥65 years, OR: 1.13, 95% CI: 1.1–1.2), Black race (OR: 1.13, 95% CI: 1.1–1.2), more than three comorbidities (OR: 1.73, 95% CI: 1.1–2.7) (Table 3), deficiency anemia (OR: 1.27, 95% CI: 1.2–1.4), congestive heart failure (OR: 1.34, 95% CI: 1.3–1.4), chronic pulmonary disease (OR: 1.43, 95% CI: 1.4–1.5), coagulopathy (OR: 1.32, 95% CI: 1.2–1.5), fluid and electrolyte disorders (OR: 1.62, 95% CI: 1.5–1.7), other neurological disorders (OR: 1.36, 95% CI: 1.3–1.5), obesity (OR: 1.60, 95% CI: 1.5–1.7), paralysis (OR: 1.11, 95% CI: 1.0–1.2), psychoses (OR: 1.14, 95% CI: 1.0–1.3), pulmonary circulation disorders (OR: 1.25, 95% CI: 1.1–1.4), and weight loss (OR: 1.20, 95% CI: 1.1–1.4) (Table 4). Protective factors identified included treatment at a hospital in the Midwest or North Central region (OR: 0.52, 95% CI: 0.5–0.6), a history of AIDS (OR: 0.21, 95% CI: 0.0–0.9), rheumatoid arthritis (OR: 0.82, 95% CI: 0.7–1.0), and hypertension (OR: 0.90, 95% CI: 0.8–1.0) (Table 4).

**Table 3:** CPAP-associated risk factors in AIS-OSA patients.

Variable	Multivariate Logistic Regression		
	OR	95% CI	*p*
Age ≥65 years old	1.125	1.049–1.206	0.001
Female	0.850	0.804–0.900	<0.001
Race			
White	Ref	–	–
Black	1.133	1.049–1.224	0.002
Hispanic	1.179	1.051–1.322	0.005
Asian or Pacific Islander	1.599	1.303–1.962	<0.001
Native American	0.961	0.643–1.437	0.847
Other	0.840	0.753–0.938	0.002
Number of comorbidity			
0	Ref	–	–
1	1.588	1.014–2.485	0.043
2	1.687	1.090–2.609	0.019
≥3	1.727	1.116–2.671	0.014
Type of insurance			
Medicare	Ref	–	–
Medicaid	1.152	1.031–1.288	0.012
Private insurance	1.082	1.003–1.167	0.041
Self-pay	0.942	0.779–1.138	0.534
No charge	0.812	0.412–1.601	0.548
Other	1.321	1.125–1.552	0.001
Bed size of hospital			
Small	Ref	–	–
Medium	1.031	0.944–1.127	0.494
Large	1.179	1.089–1.277	<0.001
Elective admission	0.812	0.750–0.878	<0.001
Teaching hospital	1.177	1.108–1.250	<0.001
Urban hospital	1.098	0.981–1.230	0.104
Region of hospital			
Northeast	Ref	–	–
Midwest or North Central	0.519	0.480–0.561	<0.001
South	0.605	0.563–0.651	<0.001
West	0.617	0.565–0.674	<0.001
Disposition			
Routine	Ref	–	–
Transfer to short-term hospital	1.817	1.576–2.094	<0.001
Transfer other	1.906	1.796–2.023	<0.001
Home Health Care (HHC)	1.585	1.467–1.713	<0.001
Against medical advice (AMA)	1.160	0.774–1.739	0.472
Died in hospital	2.944	2.641–3.281	<0.001
Discharged alive	2.895	1.138–7.362	0.026

AIDS, acquired immunodeficiency syndrome; OR, odds ratio; CI, confidence interval.

**Table 4:** Relationship between CPAP treatment and disease comorbidities.

Comorbidities	Univariate analysis			Multivariate logistic regression		
	No CPAP	CPAP	*p*	OR	95% CI	*p*
Acquired immune deficiency syndrome	115 (0.1%)	2 (0.0%)	0.041	0.21	0.05–0.87	0.031
Alcohol abuse	2,943 (3.0%)	240 (3.7%)	0.003	1.10	0.96–1.27	0.175
Deficiency anemia	12,059 (12.5%)	1,087 (16.8%)	<0.001	1.27	1.18–1.36	<0.001
Rheumatoid arthritis/collagen vascular diseases	2,945 (3.1%)	159 (2.5%)	0.007	0.82	0.70–0.97	0.017
Chronic blood loss anemia	666 (0.7%)	52 (0.8%)	0.290	0.98	0.73–1.30	0.863
Congestive heart failure	21,673 (22.5%)	2,096 (32.4%)	<0.001	1.34	1.27–1.43	<0.001
Chronic pulmonary disease	30,350 (31.4%)	2,644 (40.8%)	<0.001	1.43	1.35–1.51	<0.001
Coagulopathy	5,300 (5.5%)	557 (8.6%)	<0.001	1.32	1.20–1.45	<0.001
Depression	16,517 (17.1%)	1,078 (16.6%)	0.336	0.99	0.92–1.06	0.683
Diabetes, uncomplicated	32,699 (33.9%)	2,199 (34.0%)	0.894	1.01	0.95–1.07	0.798
Diabetes with chronic complications	19,288 (20.0%)	1,525 (23.5%)	<0.001	1.00	0.94–1.08	0.924
Drug abuse	1,874 (1.9%)	167 (2.6%)	<0.001	1.12	0.95–1.33	0.177
Hypertension	83,047 (86.0%)	5,507 (85.0%)	0.025	0.90	0.83–0.97	0.004
Hypothyroidism	15,631 (16.2%)	981 (15.1%)	0.027	0.93	0.87–1.00	0.058
Liver disease	2,418 (2.5%)	207 (3.2%)	0.001	1.03	0.88–1.19	0.737
Lymphoma	576 (0.6%)	44 (0.7%)	0.405	1.04	0.76–1.43	0.792
Fluid and electrolyte disorders	22,249 (23.0%)	2,355 (36.4%)	<0.001	1.62	1.53–1.72	<0.001
Metastatic cancer	1,085 (1.1%)	85 (1.3%)	0.166	1.01	0.81–1.28	0.908
Other neurological disorders	10,906 (11.3%)	1,115 (17.2%)	<0.001	1.36	1.27–1.46	<0.001
Obesity	37,679 (39.0%)	3,319 (51.3%)	<0.001	1.60	1.51–1.69	<0.001
Paralysis	18,460 (19.1%)	1,459 (22.5%)	<0.001	1.11	1.04–1.18	0.001
Peripheral vascular disorders	16,835 (17.4%)	1,112 (17.2%)	0.580	0.97	0.91–1.04	0.436
Psychoses	3,719 (3.9%)	299 (4.6%)	0.002	1.14	1.01–1.29	0.042
Pulmonary circulation disorders	5,779 (6.0%)	598 (9.2%)	<0.001	1.25	1.14–1.37	<0.001
Renal failure	23,161 (24.0%)	1,886 (29.1%)	<0.001	1.06	1.00–1.13	0.062
Solid tumor without metastasis	2,128 (2.2%)	164 (2.5%)	0.083	1.05	0.89–1.24	0.567
Peptic ulcer disease excluding bleeding	201 (0.2%)	17 (0.3%)	0.358	1.05	0.64–1.74	0.846
Valvular disease	10,714 (11.1%)	773 (11.9%)	0.038	0.94	0.87–1.02	0.161
Weight loss	2,962 (3.1%)	323 (5.0%)	<0.001	1.20	1.06–1.36	0.003

OR, odds ratio; CI, confidence interval.

Risk adjusted for patient characteristics and hospital characteristics, including age, sex, race, number of comorbidity, type of admission, bed size of hospital, teaching status of hospital, location of hospital, type of insurance, location of the hospital.

### Factors associated with CPAP in AIS-OSA patients

Among patients with AIS-OSA, CPAP treatment during hospitalization was associated with increased odds of developing sepsis (OR: 2.32; 95% CI: 2.1–2.6), pneumonia (OR: 2.53; 95% CI: 2.4–2.7), acute myocardial infarction (OR: 1.74; 95% CI: 1.5–1.9), deep vein thrombosis (OR: 1.81; 95% CI: 1.6–2.1), pulmonary embolism (OR: 1.58; 95% CI: 1.3–1.9), cardiac arrest (OR: 1.60; 95% CI: 1.3–2.0), acute kidney injury (OR: 1.94; 95% CI: 1.8–2.1), urinary tract infection (OR: 1.42; 95% CI: 1.3–1.5), intracranial hemorrhage (OR: 1.57; 95% CI: 1.4–1.8), receipt of thrombolysis (OR: 1.29; 95% CI: 1.1–1.5), and receipt of mechanical thrombectomy (OR: 1.41; 95% CI: 1.2–1.7) (Table 5).

**Table 5:** Relationship between CPAP treatment and complications.

Complications	Univariate analysis			Multivariate logistic regression		
	No CPAP	CPAP	*p*	OR	95% CI	*p*
Medical complications						
Sepsis	3,789 (3.9%)	561 (8.7%)	<0.001	2.32	2.12–2.55	<0.001
AMI	4,676 (4.8%)	527 (8.1%)	<0.001	1.74	1.58–1.91	<0.001
Deep vein thrombosis	1,618 (1.7%)	194 (3.0%)	<0.001	1.81	1.56–2.11	<0.001
pulmonary embolism	977 (1.0%)	103 (1.6%)	<0.001	1.58	1.29–1.94	<0.001
Cardiac arrest	986 (1.0%)	105 (1.6%)	<0.001	1.60	1.30–1.96	<0.001
AKI	15,087 (15.6%)	1,713 (26.5%)	<0.001	1.94	1.83–2.06	<0.001
Pneumonia	7,000 (7.3%)	1,069 (16.5%)	<0.001	2.53	2.36–2.71	<0.001
UTI	8,661 (9.0%)	795 (12.3%)	<0.001	1.42	1.31–1.53	<0.001
Neurological complications						
Seizure	4,011 (4.2%)	299 (4.6%)	0.072	1.12	0.99–1.26	0.073
Intracranial hemorrhage	3,068 (3.2%)	319 (4.9%)	<0.001	1.57	1.40–1.77	<0.001
Thrombolysis	2,557 (2.6%)	219 (3.4%)	<0.001	1.29	1.12–1.48	<0.001
MT	1,637 (1.7%)	154 (2.4%)	<0.001	1.41	1.20–1.67	<0.001
Die in hospital	3,325 (3.4%)	446 (6.9%)	<0.001	2.07	1.87–2.30	<0.001
LOS	6 (3–11)	4 (2–7)	<0.001	2.68	2.55–2.82	<0.001
TOTCHG	62,003 (31,820–124, 986)	37,726 (21,830–1,796)	<0.001	2.41	2.29–2.54	<0.001

OR, odds ratio; CI, confidence interval; AKI, acute kidney injury; UTI, urinary tract infection; MT, mechanical thrombectomy; AMI, acute myocardial infarction.

## Discussion

A large international case–control study demonstrated a statistically significant association between sleep impairments and the risk of acute stroke (17, 18). OSA is a well-established risk factor for AIS (18). A meta-analysis revealed that OSA is present in up to 70% of stroke and transient ischemic attack patients (19). Despite its high prevalence, only a small proportion of stroke patients undergo OSA testing and receive treatment (20). According to a cross-sectional study using NIS data, the documented incidence of OSA in acute cerebral infarction patients was 3.1% (21). In our retrospective analysis, we identified 103,004 hospitalized AIS-OSA patients between 2010 and 2019, of whom 6,476 (6.3%) received CPAP treatment (Figure 2). Among AIS-OSA patients who received CPAP treatment, most of patients (75.4%) were treated for less than 24 h (Figure 3). However, these aggregate treatment durations do not reflect patients’ adherence to CPAP and are therefore uninformative for assessing therapeutic efficacy (22). As administrative codes do not capture why CPAP was withheld, its absence in the non-CPAP group could reflect patient intolerance, refusal, contraindications, or unavailability of equipment during hospitalization, but these reasons cannot be ascertained from the NIS.

While a correlation exists between OSA and stroke, it remains unclear whether varying demographic characteristics or clinical factors influence outcomes in patients with AIS-OSA (23). CPAP is the gold standard for OSA treatment, though its efficacy in AIS-OSA patients remains uncertain (24, 25). Understanding the risk factors affecting CPAP treatment in AIS-OSA patients is crucial for effective disease management.

In this study, patients with AIS-OSA who received CPAP exhibited higher in-hospital mortality, longer LOS, greater TOTCHG, and a different distribution of discharge dispositions (Table 2). These findings were consistent with reports from other national hospital discharge databases (26–29). We also found that AIS-OSA patients treated with CPAP had more than three comorbidities (Table 2). These comorbidities (e.g., sepsis, heart failure, pneumonia) were pre-existing conditions present before the initiation of CPAP treatment, reflecting a greater baseline burden of illness. Compared to non-CPAP-treated patients, those receiving CPAP had higher in-hospital mortality, longer LOS, and greater TOTCHG. However, among surviving CPAP-treated patients, a statistically significantly higher proportion were discharged routinely (Table 2), suggesting that CPAP may be associated with better prognoses and improved neurological recovery at discharge in this subgroup. This paradox also may reflect selection and survivorship biases: patients able to tolerate or be offered CPAP may have been less neurologically impaired at baseline (we lacked NIHSS or GCS data), whereas the most severe cases, who could not tolerate or were never selected for CPAP, died earlier and were thus excluded from the denominator of survivors eligible for discharge. OSA can induce hypercapnia, which paradoxically reduces blood flow to ischemic brain regions while increasing perfusion to unaffected cerebral vessels (30). In AIS patients, inadequate perfusion of the ischemic penumbra leads to neurological damage and functional decline (31, 32). CPAP treatment may help prolong the survival time of the ischemic penumbra, promote neuronal functional recovery, and reduce the risk of neurological deterioration. Furthermore, CPAP improves sleep quality and oxygen saturation by providing pressure support to maintain upper airway patency during sleep (33). It also exerts multifaceted effects on stroke pathophysiology, including modulation of neuronal apoptosis, mitochondrial bioenergetics, oxidative stress, angiogenesis, glucose metabolism, and blood–brain barrier regulation (34, 35).

**Figure 4:**
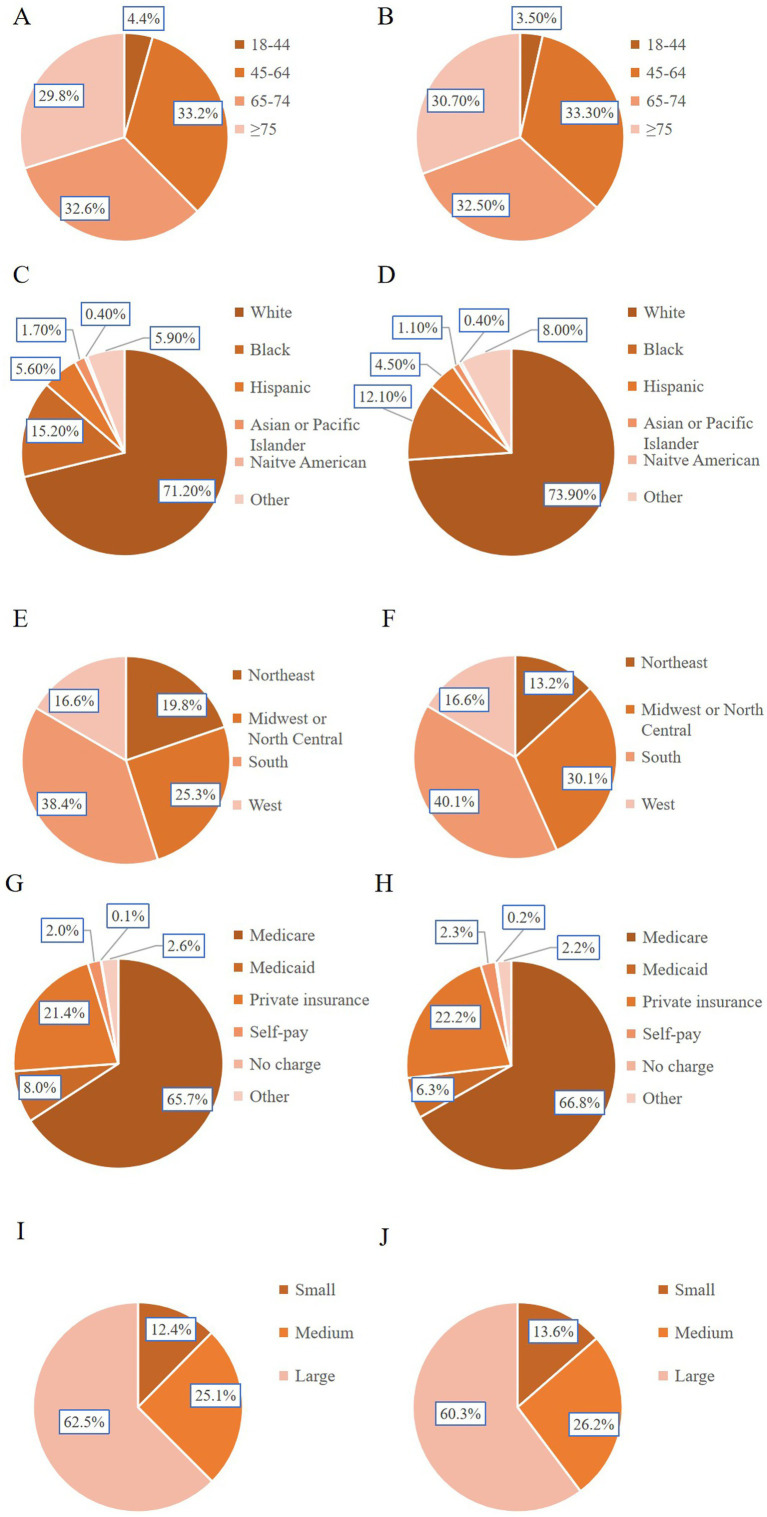
Patient demographics and hospital characteristics between the two groups. **(A)** Age distribution analysis of CPAP patients. **(B)** Analysis of age distribution of patients without CPAP. **(C)** Racial distribution analysis of CPAP patients. **(D)** Racial distribution analysis of patients without CPAP. **(E)** Analysis of hospital regional distribution of CPAP patients. **(F)** Analysis of hospital regional distribution of patients without CPAP. **(G)** Analysis of Insurance Types for CPAP Patients. **(H)** Analysis of Insurance Types for Patients without CPAP. **(I)** Analysis of the number of hospital beds for CPAP patients. **(J)** Analysis of the number of hospital beds for patients without CPAP.

Our investigation revealed older age (≥ 65 years) and male sex as independent risk factors for CPAP treatment (Figures 4A,B). While OSA can manifest across all ages, its prevalence increases with age, tending to plateau after approximately 65 years (36). A significant predominance of OSA in men has been observed (37), with hormonal influences on breathing control and upper airway muscle activation during sleep, as well as sex-specific fat distribution, appearing to contribute to this disparity (38, 39). The analysis indicated a higher incidence of CPAP treatment among Black, Hispanic, and Asian or Pacific Islander individuals (Figures 4C,D). The African American race is associated with untreated OSA and serves as an independent predictor for stroke and elevated all-cause mortality, even after adjusting for cardiovascular risk factors, compared to White individuals (40). The prevalence of diagnosed OSA is notably elevated among racial/ethnic minorities, and rates of undiagnosed OSA are particularly high within these groups (41). Factors such as a higher likelihood of residing in economically deprived neighborhoods, statistically significantly greater rates of obesity, and increased prevalence of hypertension (42) contribute to the severity of OSA and influence CPAP usage, consistent with other studies. Glaucylara et al. (43) reported increased levels of cardiovascular risk factors associated with OSA, including elevated neutrophil counts, an inflammatory marker, which was strongly associated with men, younger individuals, and African American individuals. One study suggested that OSA patients exhibiting phenotypes associated with age, gender, BMI, and sleep apnea severity demonstrate slightly higher CPAP usage (44).

Our study revealed a higher likelihood of receiving CPAP treatment in northeastern hospitals (Figures 4E,F), a finding consistent with previous literature. Levi Dunietz et al. reported statistically significant state-level and regional disparities in both CPAP treatment and adherence among Medicare beneficiaries with OSA suggest gaps in delivery of OSA care for Americans (45). These gaps may stem from demographic differences, including racial variation, as well as from socioeconomic status, urbanicity, and access to accredited sleep centers (45). Patients receiving CPAP were more likely to be Medicaid beneficiaries (Figures 4G,H), possibly due to the higher healthcare costs associated with CPAP-treated patients (28, 29) and their increased likelihood of belonging to racial/ethnic minority groups residing in economically deprived regions (41, 42). CPAP treatment was also more prevalent in large hospitals compared to small ones (Figures 4I,J), potentially attributable to the complexity of care and the higher patient volume in larger institutions (46).

**Figure 5:**
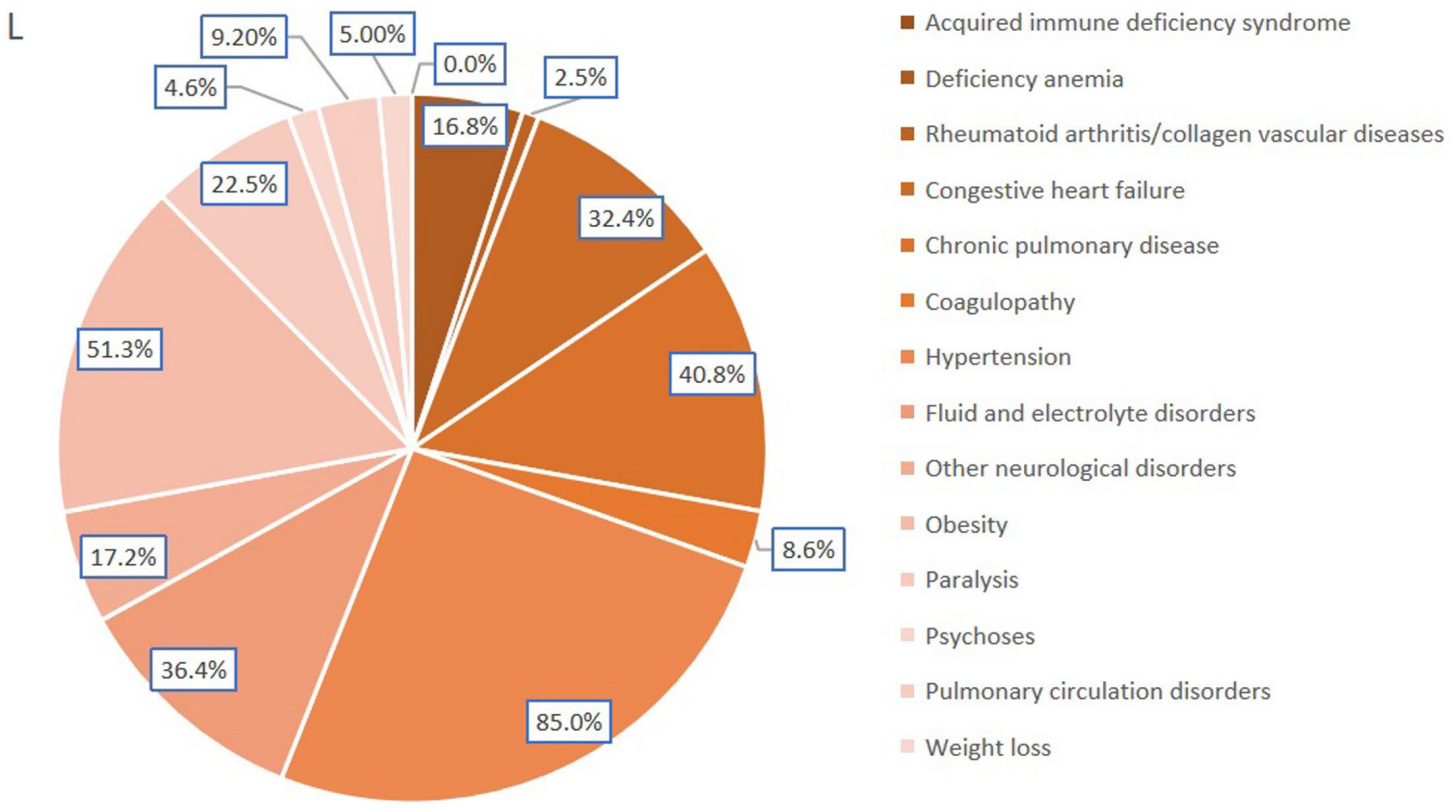
Incidence of CPAP-associated comorbidities in AIS-OSA patients.

In this study, acquired immune deficiency syndrome, deficiency anemia, congestive heart failure, chronic pulmonary disease, coagulopathy, fluid and electrolyte disorders, other neurological disorders, paralysis, psychoses, pulmonary circulation disorders, obesity, alcohol abuse, drug abuse, and weight loss were associated with CPAP treatment; however, these associations do not imply causation (Figure 5). Given the interconnected nature of stroke and sleep apnea, with numerous shared risk factors and comorbidities, it remains uncertain at an individual level whether sleep apnea is a potential cause or a consequence of stroke (3, 6). Obesity, pulmonary circulation disorders, hypertension, diabetes, and other comorbidities are consistently reported in patients with stroke diagnosed with obstructive sleep apnea (5, 47). All these factors influence the severity of the disease and, consequently, CPAP treatment. In cases of severe OSA, treatment is essential, and CPAP is the recommended first-line therapy (38).

**Figure 6:**
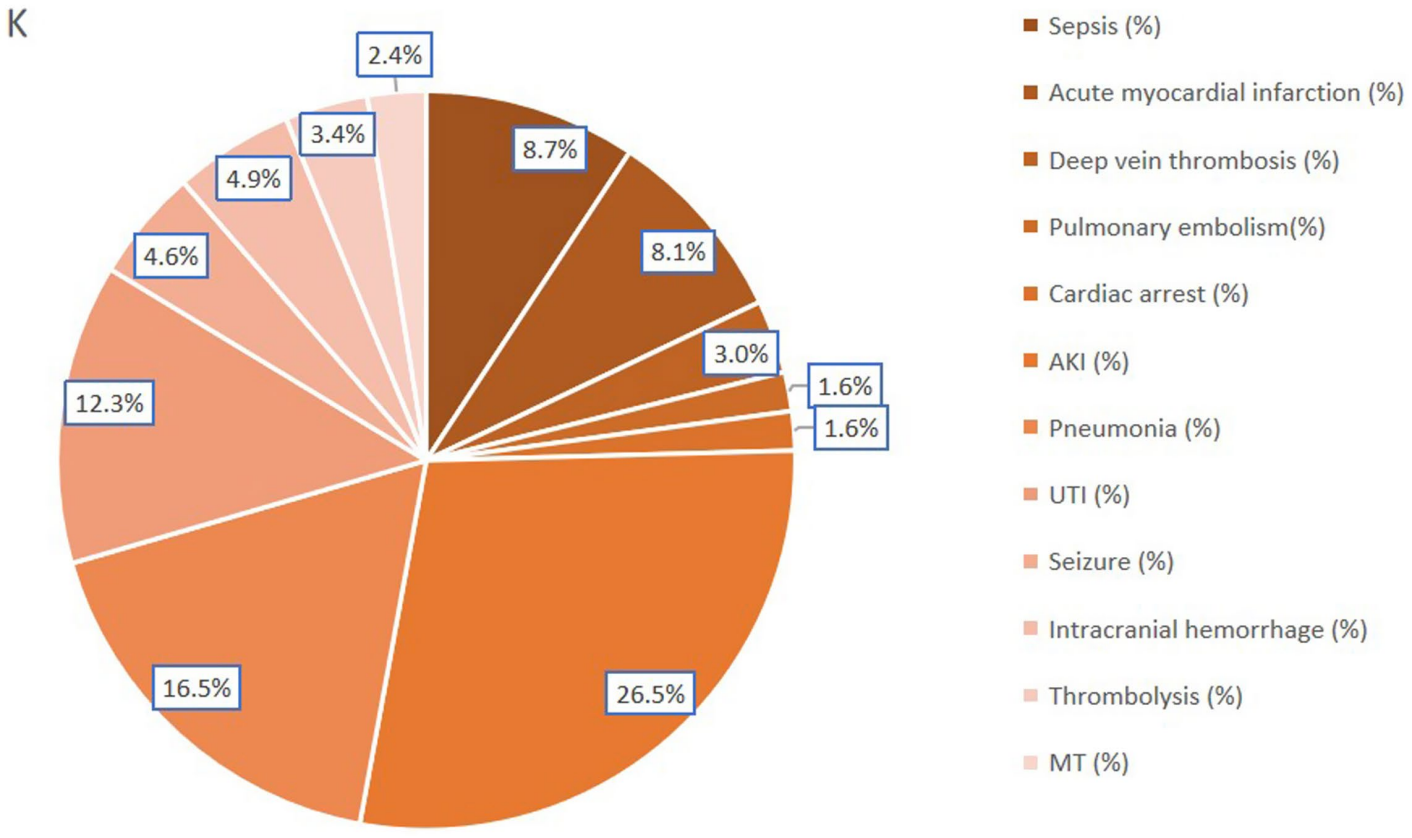
Incidence of CPAP-associated complications in AIS-OSA patients.

Patient-related factors associated with CPAP treatment in our study included sepsis, pneumonia, acute myocardial infarction, deep vein thrombosis, pulmonary embolism, cardiac arrest, acute kidney injury, urinary tract infection, intracranial hemorrhage, thrombolysis, and mechanical thrombectomy (Figure 6). Prior research has indicated that prolonged mechanical ventilation is associated with a high incidence of thrombosis in critically ill patients despite prophylaxis (48). While a prospective randomized clinical trial (49) found that CPAP reduced early pulmonary infection rates and endotracheal intubation rates, our study demonstrated that CPAP treatment was associated with an increased incidence of lung infection. This discrepancy may be attributable to the possibility that CPAP-treated patients in our cross-sectional study presented with more complications, more severe underlying conditions, and a higher predisposition to developing lung infections. Our findings suggest that AIS patients receiving CPAP treatment were more likely to undergo mechanical thrombectomy or thrombolysis and experience acute myocardial infarction or cardiac arrest, potentially due to OSA’s capacity to induce pathological cardiac and cerebrovascular effects (50), and the potential for ischemic stroke lesions in critical areas regulating airway patency or breathing effort to exacerbate sleep apnea (3).

The relationship observed between CPAP treatment and complications is associative rather than causal. Establishing a definitive causal link between CPAP and negative outcomes is inherently challenging due to the methodological limitations of observational and database studies. Patients receiving CPAP treatment frequently present with a higher burden of comorbidities or postoperative complications, which may represent the true underlying causes of adverse outcomes, rather than the CPAP treatment itself. While efforts are made to adjust for these confounding factors, a direct causal relationship cannot be definitively established. Notably, a meta-analysis of randomized trials indicated that early CPAP treatment demonstrated no effect, whereas CPAP treatment initiated beyond 7 days showed beneficial effects (11). Given that conventional CPAP treatment may not invariably be beneficial and could potentially have adverse effects in specific patient populations, it is exceedingly important to consider a personalized approach to OSA treatment in AIS patients.

Further research is warranted to delineate the clinical characteristics of patients who would and would not derive benefit from CPAP treatment. Moreover, large-scale prospective trials are necessary to rigorously evaluate the risks and benefits of routine versus more selective CPAP application among patients with OSA in the setting of hospitalized AIS in general. Should these findings be corroborated in randomized prospective studies, a more selective treatment paradigm will be essential to improving mortality rates, reducing length of hospital stay, decreasing complications, and lowering associated healthcare expenditures.

While our findings offer valuable insights, several limitations inherent in our analysis warrant consideration. Firstly, our identification of OSA-AIS patients between 2010 and 2019 relied on ICD-9-CM/ICD-10-CM codes. This approach lacked comprehensive data regarding disease severity, as well as scores from the National Institutes of Health Stroke Scale (NIHSS), Glasgow Coma Scale (GCS), and Modified Rankin Scale (MRS). Without these metrics we were unable to adjust for baseline neurological impairment, which is a strong predictor of mortality and functional outcome. To partially address this limitation, we included the comorbidity “other neurological disorders” as a proxy indicator of pre-existing neurological disease burden; nevertheless, this surrogate cannot fully capture acute stroke severity, and residual confounding may remain. Furthermore, because the database contains no information on the timing of CPAP initiation (early versus late), actual adherence during hospitalization, or any pre-admission CPAP treatment, we cannot separate the true effect of CPAP from these unmeasured factors (10, 51), any observed associations may therefore reflect timing, adherence, or prior exposure rather than the treatment itself. Secondly, our analysis is further constrained by the absence of device-specific information in the NIS. Knowing whether patients received fixed-level or auto-titrating CPAP (the actual pressure settings) is essential for interpreting any outcome differences (52). Moreover, the beneficial effects of CPAP are highly dependent on adequate adherence: prior work has shown that nightly use of ≥4 h was considered adherent (53). Because the NIS does not record mask-on time or device-download data, we cannot determine whether the observed associations were diluted by sub-therapeutic or intermittent in-hospital use. Thirdly, the inherent challenges associated with incomplete or inaccurate database records and the absence of randomization, characteristic of our study’s retrospective design, introduce a higher risk of unidentifiable confounding factors that could influence the observed results. Fourthly, our study data were derived from the NIS, a United States in-hospital database, which may introduce selection bias due to the exclusion of data from outpatient, nursing home, and global settings. While our larger sample size helps to mitigate the impact of these errors, these limitations should be acknowledged. Despite these constraints, the consistency of our results with previous studies lends further credence to our findings.

## Conclusion

Our findings indicate that hospitalized AIS-OSA patients treated with CPAP experienced increased in-hospital mortality, LOS, and TOTCHG. However, among the surviving CPAP-treated patients, a statistically significantly higher proportion experienced routine discharge. This observation may suggest that surviving patients who received CPAP treatment had more favorable prognoses. We also identified independent predictors and associated factors for CPAP treatment in hospitalized AIS-OSA patients. Randomized controlled trials specifically focusing on AIS-OSA patients within various high-risk groups are necessary to refine the current consensus regarding the initiation of CPAP in the acute setting.

## Data Availability

The datasets presented in this study can be found in online repositories. The names of the repository/repositories and accession number(s) can be found at: https://www.hcup-us.ahrq.
